# Nodding Syndrome May Be Only the Ears of the Hippo

**DOI:** 10.1371/journal.pntd.0003880

**Published:** 2015-08-13

**Authors:** Joseph Francis Wamala, Mugagga Malimbo, Floribert Tepage, Luswa Lukwago, Charles Lukoya Okot, Robert O. Cannon, Anne Laudisoit, Robert Colebunders

**Affiliations:** 1 National Disease Control, Ministry of Health, Kampala, Uganda; 2 National Onchocerciasis Control Program, Ministry of Health, Kisangani, Democratic Republic of the Congo; 3 Disease Prevention and Control Cluster, World Health Organization, Kampala, Uganda; 4 ROC Consulting, Palo Alto, California, United States of America; 5 Evolutionary Biology Group, University of Antwerp, Antwerp, Belgium; 6 Institute of Integrative Ecology, University of Liverpool, Liverpool, United Kingdom; 7 Department of Epidemiology and Social Medicine, University of Antwerp, Antwerp, Belgium; 8 Department of Clinical Sciences, Institute of Tropical Medicine, Antwerp, Belgium; Federal University of Agriculture, NIGERIA

## Introduction

Nodding Syndrome (NS) is a neurological disorder of unknown etiology and pathogenesis, reported to occur mainly in the Western Equatoria State in South Sudan, in several districts in northern Uganda, and in Mahenge district in Tanzania [1].

The characteristic clinical feature of NS is a paroxysmal spell where the head nods forward repeatedly, 5–20 times per minute, in a seemingly unresponsive affected child. These nodding episodes represent an atonic form of epilepsy during which generalized electrodecrement is seen on electroencephalography and paraspinal dropout on electromyography [2]. During these episodes, children exhibit differing levels of decreased awareness, confusion, and unresponsiveness to commands [1]. Children with NS develop varying degrees of mental retardation, and in some, there is considerable stunting of growth and failure to develop secondary sexual characteristics. The syndrome generally appears between the ages of five and 18. Affected children are generally reported to be healthy until the nodding episodes begin. The natural history is not well known but has been described as starting with nodding and often progressing to generalized tonic-clonic seizures with accumulated sequelae. No proven effective specific treatment is available, and many children have died as a result of uncontrolled seizures that led to drowning or burning [1]. Recent studies, however, show that children with NS who receive adequate care—including anti-epileptic treatment, nutritional and emotional support, and physical rehabilitation—may substantially improve clinically [3].

NS is characterized by temporal, geographical, and familial clustering in villages in some onchocerciasis-endemic areas. The extent of the outbreaks in South Sudan and northern Uganda has made NS a major public health problem in these countries. In contrast, in Tanzania, the prevalence of NS is low and stable [4]. NS, like other forms of epilepsy, is associated with social stigma and can have severe socioeconomic consequences for families and communities.

Various infectious, toxic, nutritional, psychosocial, and genetic causes for NS have been proposed, but none have been confirmed [5]. Cerebrospinal fluid (CSF) total protein, cells, and glucose are generally normal [2]. In a study in Uganda of 19 patients with NS, brain MRIs without contrast, performed using a 0.5 Tesla machine, showed different degrees of cortical and cerebral atrophy but no focal changes [6]. However, in a study of 12 patients with NS in Tanzania, using an MRI machine with better resolution (1.5 Tesla), five had gliotic lesions and five had changes in the hippocampus [7]. A weak association between *Onchocerca volvulus* (OV) skin PCR positivity and lesions on MRI was reported [7]. No autopsy or brain biopsy data for NS patients has been published.

Epidemiological studies suggest a strong association between NS and onchocerciasis [8]. NS is only known to occur in onchocerciasis-endemic areas, and case-control studies have demonstrated a statistically significant higher prevalence of onchocerciasis in individuals with NS than in controls [7,9,10]. It is, however, unclear how onchocerciasis might cause NS because microfilariae (mf) and adult OV worms are not generally considered able to invade the central nervous system.

In 1976, Duke et al., however, noted the presence of small numbers of OV microfilariae in the CSF (<2 mf/ml) in five of eight untreated, heavily infected (>100 mf/mg skin) onchocerciasis patients. During diethylcarbamazine treatment, in 10 of 11 heavily infected patients presenting with an ocular form of onchocerciasis, the numbers of OV microfilariae in the CSF increased even up to 8–31 mf/ml [11]. However, more recently, a Tanzanian study of patients with onchocerciasis and epilepsy did not have microfilariae in the CSF, and PCR of CSF of patients with NS failed to identify OV DNA [7,10,12]. The latter results are difficult to interpret, as it was unclear how many participants had been previously treated with ivermectin and how frequently [13]. Most of the patients (60.8%) had visible microfilariae on microscopic examination of the skin biopsies, but the mean microfilariae density was low, 3.6 mf/mg, potentially because of prior ivermectin treatment [12].

## Hypothesis

We hypothesize that (1) NS is part of a spectrum of different types of seizures in onchocerciasis-endemic areas, and that (2) several of the different types of epilepsy most likely have a related etiology as a direct and/or indirect (potentially immune-mediated) consequence of OV infection.

## These Hypotheses Are Based on the Following Arguments

In areas where NS is very prevalent, different types of seizures—both NS and convulsive epilepsy without NS—often occur among children within the same family [14].

**Fig 1:**
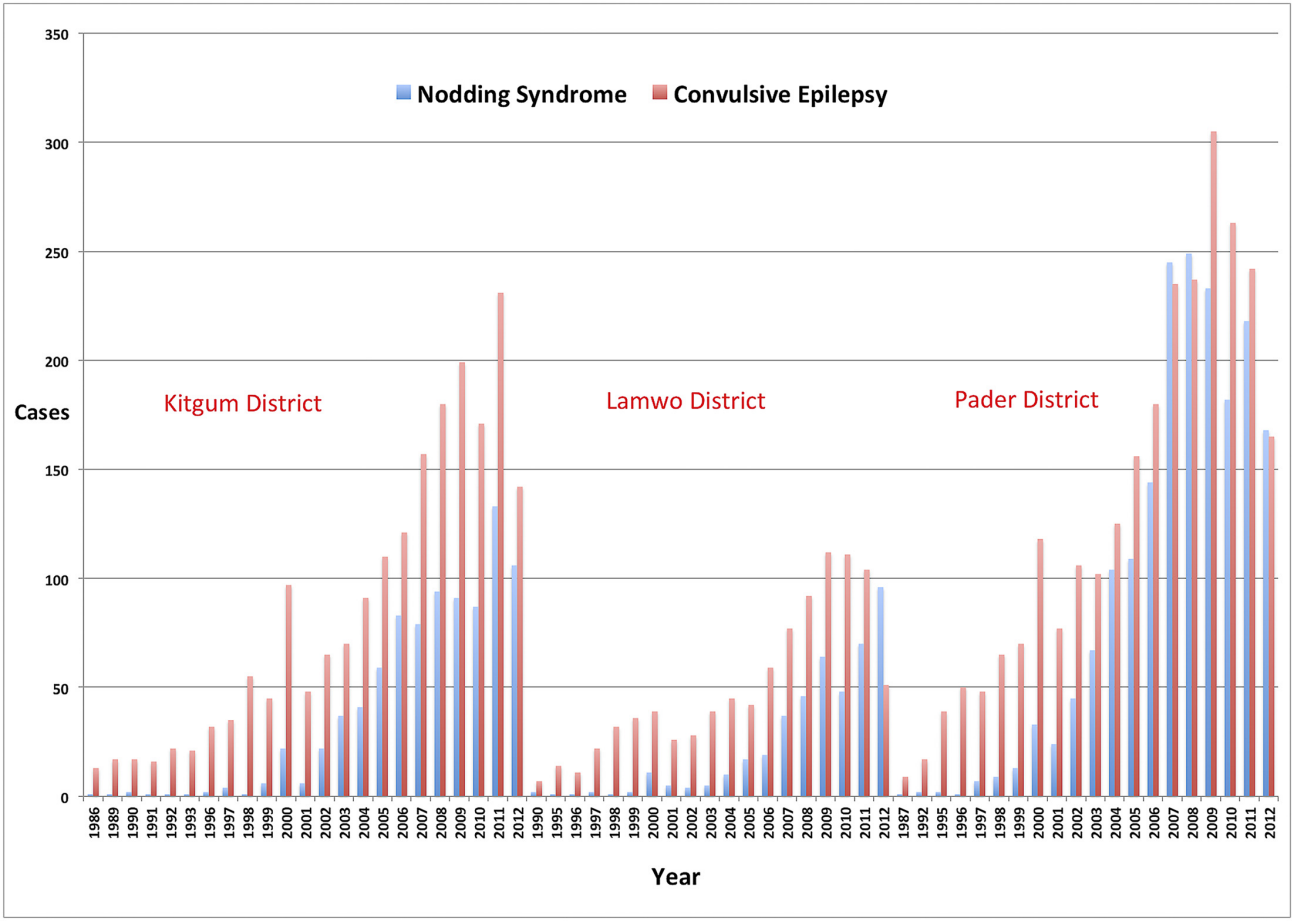
Number of suspected cases of Nodding Syndrome and convulsive epilepsy, by year of onset in Kitgum, Lamwo, and Pader Districts, Uganda (census of June 2012).

A house-to-house census of NS and convulsive epilepsy, conducted by trained village health teams from the Ugandan Ministry of Health in June 2012 in northern Uganda [15], provides evidence that the NS outbreak in Uganda occurred simultaneously with a convulsive epilepsy outbreak in the same districts, with more cases of convulsive epilepsy than NS (Fig 1). Households were asked about family members with NS and other epilepsy symptoms. A suspected case of NS was defined, according to the 2012 WHO case definition of NS, as a person with reported head nodding (repetitive involuntary drops of the head towards the chest on two or more occasions in a previously normal person [16]). For every suspected case of NS and convulsive epilepsy, the year of onset of the symptoms was noted. During a subsequent NS prevalence study performed in March 2013, several suspected NS cases identified during the 2012 survey, upon reevaluation, were no longer considered as NS cases, thereby increasing the number of non-NS epilepsy cases in the affected districts [15].

**Fig 2:**
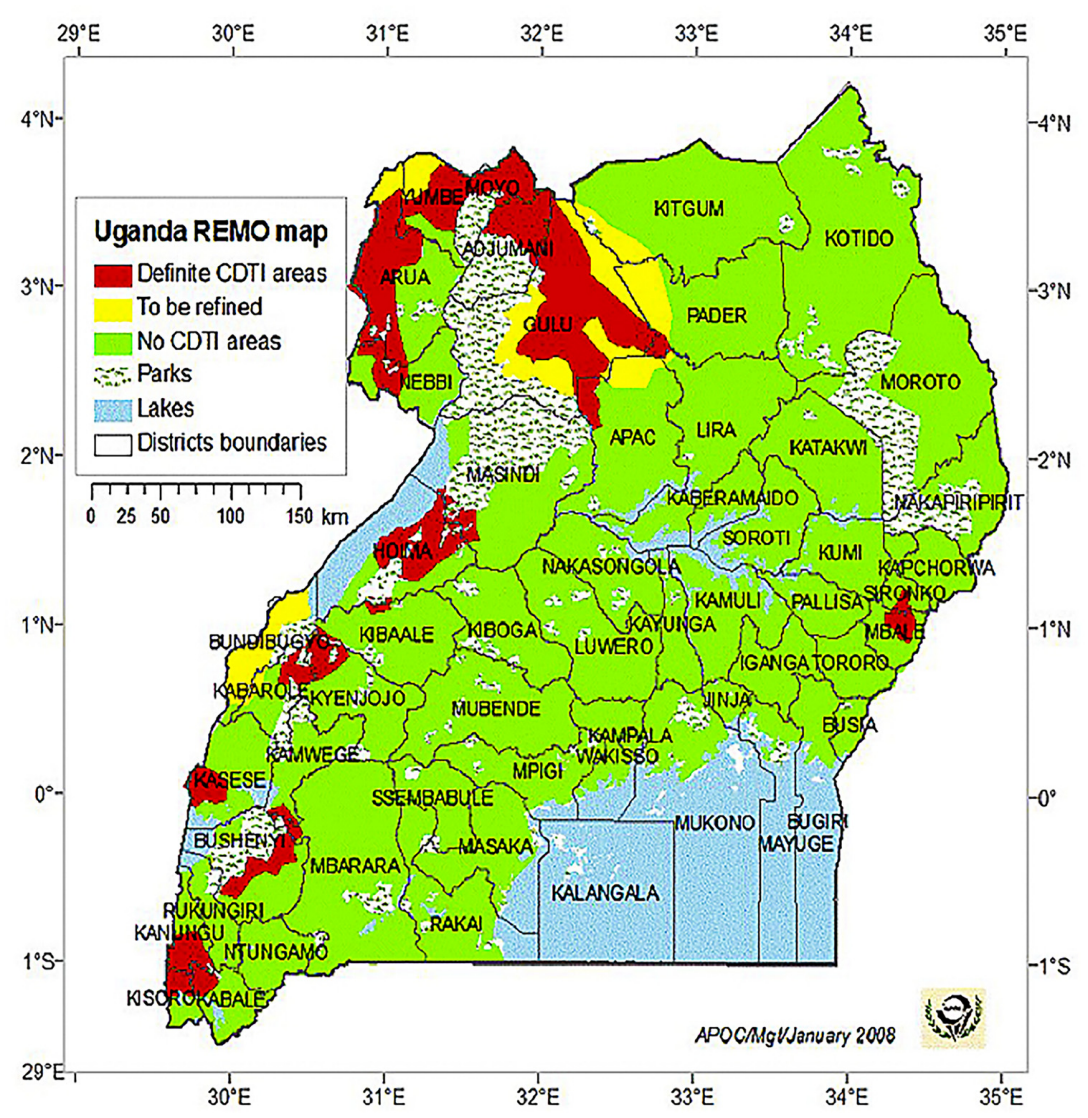
Rapid epidemiological map of onchocerciasis (REMO) (www.who.int/apoc/cdti/remo/) showing that in 2008, in the Kitgum and Pader Districts, Community Directed Treatment with Ivermectin (CDTI) was not available.

In Kitgum and Pader, the most NS-affected districts of Uganda because of the war in those districts, Community Directed Treatment with Ivermectin (CDTI) was not available before 2009 (Fig 2) and was implemented in some of the most affected villages only in 2012. Since ivermectin has been distributed biannually in NS-affected districts in northern Uganda and larviciding major rivers (targeting the blackfly vector of OV) has been implemented, there has been a dramatic drop in the number of new NS cases. There were no officially reported cases in 2013 [17], and only two new NS cases were detected from 2014 to the present (personal communication, B Opar to JF Wamala. See Acknowledgments). While this is not proof that the drop was caused by widespread CDTI and/or by larviciding rivers, it is highly suggestive because, in South Sudan, where the coverage of ivermectin has always been very low and larviciding rivers is currently not done, new cases of NS continue to appear (personal communication, S Komyangi to R Colebunders. See Acknowledgments). Whether there has been a similar decline of other forms of epilepsy in northern Uganda needs further investigation. The experience in Uganda is a convincing argument against the possibility that ivermectin could be causing cases of NS.

Higher convulsive epilepsy prevalence rates have been observed in many areas endemic for onchocerciasis [18]. Based on a meta-analysis of eight population-based surveys performed in seven different countries, Pion et al. calculated that, on average, the prevalence of epilepsy increased by 0.4% for each 10% increase in onchocerciasis prevalence [18]. Moreover, NS-like features have been reported in many onchocerciasis-endemic areas, e.g., in Liberia, Burundi, Ethiopia, Cameroon, and Mali [19]. In a study in Cameroon, the closer a village was to the Mbam River (a known habitat of the blackfly vector of OV), the higher the prevalence of epilepsy. In addition, the prevalence of epilepsy and community microfilarial load (microfilariae per skin snip) were closely related, and a case-control study demonstrated that the microfilarial loads in the epileptic group were significantly higher than in the control group [20]. The peak incidence of epilepsy in onchocerciasis-endemic regions between the ages of 10 and 15 years can be explained by the equally high incidence of OV infection in these age groups [9]. In June 2014, during a house-to-house survey in Titule, bas Uélé—an onchocerciasis-endemic area in the Oriental Province of the Democratic Republic of the Congo (DRC)—we observed an epilepsy prevalence of 2.3% [21]. This is about twice the prevalence of epilepsy observed in Africa in non–onchocerciasis-endemic areas [22]. Cases of confirmed NS meeting the 2012 WHO case definition criteria [16], were not observed during a small scale survey, but a few adolescents with epilepsy, mental retardation, important stunted growth, and absence of secondary sexual characteristics were observed, suggesting that NS may also occur occasionally [21]. Proximity of households to blackfly (Diptera: Simuliidae)-infested rivers was a risk factor for epilepsy [21]. Ivermectin distribution was first instituted in the Mbam valley in 1998 and in Titule in 2002. Therefore, since ivermectin distribution has been consistent over time, current community microfilariae load in these countries is probably not high enough to cause a large number of children to manifest a severe form of NS. In Mahenge District, Tanzania, where ivermectin distribution started in 1997 but in an erratic way [10], NS prevalence is not only much lower than in Uganda and South Sudan, but NS manifestations seem to be less clinically severe as well [6]. In the Western Equatoria state in South Sudan, although the distribution of ivermectin started in 1996, the many treatment interruptions in its subsequent use, during periods of conflict (personal communication, S Komyangi to R Colebunders. See Acknowledgments), are presumed to have facilitated the NS epidemic.

Recently, Johnson et al. demonstrated that serum autoantibodies against leiomodin-1 (a protein present in the human brain) are more likely to be present in NS cases than in controls [23]. These antibodies were also present in the cerebrospinal fluid of certain patients with NS, and were found to be neurotoxic in vitro and cross-reacting with OV-specific proteins [23]. Whether these antibodies are instrumental in the pathogenesis of NS and epilepsy and/or are a consequence of damage to the central nervous system caused by OV (or an OV endosymbiont such as *Wolbachia*, or an unknown neurotropic agent transmitted by blackflies) needs to be investigated.

## Discussion

The total number of NS cases in South Sudan, Uganda, and Tanzania is unknown but is relatively small, particularly when using the restrictive 2012 World Health Organization NS case definition [15]. In March 2013 in Uganda, the second most NS-affected country, the estimated number of children with probable NS in the three northern Ugandan districts was 1,687 [15].

NS, however, may only represent the “ears of the hippo”—when viewed from the shore with the hippo submerged in a river up to its ears—considering the “hippo” as all epilepsy cases resulting from OV infection. NS may be a very severe form of a disease that manifests in children because of their susceptibility to a pathogenic mechanism arising from the combination of both an immature nervous and immune system, and a very high exposure to OV (or byproducts of dying and dead microfilariae). Other children, less exposed to OV and/or exposed later in life, may develop tonic-clonic epilepsy with minimal or no mental retardation and no decrement of growth or sexual development.

If OV infection results in either direct and/or indirect (immune-mediated) central nervous system damage that is epileptogenic, one would expect that all onchocerciasis-endemic regions above a certain threshold of community microfilarial load would have an increased prevalence of epilepsy [20]. It is also likely, consistent with our observation in the DRC, that in many other onchocerciasis-endemic regions with insufficient ivermectin coverage, a moderately increased prevalence of epilepsy might have remained unrecognized because prevalence studies were never performed.

Initially, the NS epidemiological parameters in South Sudan and northern Uganda were similar [1,24]. The major difference in the current incidence of NS between the regions, therefore, strongly suggests that either the distribution of ivermectin or the larviciding of rivers, or a combination of both interventions, had an impact on the epidemic. It is now critically important to evaluate whether this “inadvertent experiment” can be reproduced in well-designed studies elsewhere.

## Conclusion

In this paper, we argue in favor of expanding the research on NS to include the study of all forms of epilepsy in onchocerciasis-endemic regions and to investigate in well-designed intervention studies whether ivermectin, with or without larviciding rivers, is able to decrease the incidence of epilepsy in onchocerciasis-endemic regions.

The African Programme for Onchocerciasis Control estimates there are currently 36 million people with onchocerciasis [25]. Therefore, if 1% (equivalent to the approximate excess prevalence over non-endemic areas [22]) were to develop epilepsy, the number of excess cases of epilepsy due to onchocerciasis would be on the order of 360,000. We hypothesize that most of this excess in prevalence of epilepsy is potentially preventable by increasing the coverage of ivermectin treatment and by maintaining it over many years. If it is confirmed that OV increases the risk of epilepsy, this will be an additional argument to strengthen onchocerciasis elimination plans.
